# How stress influences aggressive behavior: anxiety as a mediator and the joint moderating roles of music preferences and perceived teacher legitimacy

**DOI:** 10.3389/fpsyg.2026.1746277

**Published:** 2026-04-10

**Authors:** Lele Jiang, Ke Qi, Shuhui Xu

**Affiliations:** 1 School of Music, Wenzhou University, Wenzhou, China; 2 Department of Psychology, Wenzhou University, Wenzhou, China

**Keywords:** aggressive behavior, anxiety, moderated mediation, music preferences (upbeat & conventional), perceived teacher legitimacy, stress

## Abstract

**Background:**

Stress is closely linked to aggression, but the mechanisms underlying this association and the roles of potential protective factors remain unclear. This study examined whether anxiety statistically mediates the association between stress and aggressive tendencies, and whether music preference and perceived teacher legitimacy shape this process.

**Methods:**

A sample of 488 university students completed online measures of stress, anxiety, aggression, preference for “Upbeat and Conventional” music, and perceived teacher legitimacy. Moderated mediation analyses were conducted using Hayes' PROCESS macro with 5,000 bootstrap resamples.

**Results:**

Stress was positively associated with aggression both directly and indirectly through anxiety. Preference for “Upbeat and Conventional” music significantly moderated both the direct stress-aggression association and the anxiety-aggression association. Specifically, higher music preference was associated with a stronger direct stress-aggression link but a weaker anxiety-aggression link. Perceived teacher legitimacy was negatively associated with aggression, but its interaction with anxiety was not statistically significant.

**Conclusion:**

Anxiety statistically mediated the association between stress and aggression. Music preference showed a mixed moderating pattern, whereas perceived teacher legitimacy appeared to function primarily as a broader contextual protective factor. These findings underscore the relevance of both intra-individual and social-contextual factors in understanding aggressive tendencies.

## 1 Introduction

### 1.1 Stress and aggressive

Stress refers to the psychological and physiological response that arises when environmental demands exceed an individual's available coping resources (Lazarus, 1974). When individuals appraise external demands as surpassing their coping

capacity, high-arousal negative emotions such as anxiety and anger are often elicited, potentially leading to maladaptive behavioral responses (Pearlin et al., 1981). Among adolescents and college students, multiple stressors, including academic and interpersonal demands, are consistently associated with aggressive behavior (Jun and Choi, 2015; Fu et al., 2022).

The mechanisms linking stress to aggression primarily involve three pathways. First, the affective arousal pathway, where stress-induced negative emotions directly facilitate reactive aggression (Anderson and Bushman, 2002). Second, the cognitive control pathway, in which stress impairs prefrontal executive functions, reducing the capacity for impulse regulation (Arnsten, 2015). Third, the social information processing pathway, where stress heightens attentional bias toward threat cues and activates aggressive cognitive scripts (Huesmann and Guerra, 1997). Additionally, personality traits and environmental factors may moderate these processes, jointly shaping the expression of aggressive behavior (Hou et al., 2017).

Based on this theoretical and empirical foundation, the present study proposes:

*Hypothesis 1:* Stress positively predicts aggressive tendencies.

### 1.2 The mediating role of anxiety

Extensive evidence indicates that stress elevates aggressive tendencies primarily through emotional and cognitive processes (Anderson and Bushman, 2002; Huesmann, 1988). High stress elicits anxiety, a high-arousal negative state that activates the sympathetic and endocrine systems and impairs prefrontal executive control, reducing inhibition of impulsive and hostile responses (Lazarus, 1974; Arnsten, 2015; Cohen et al., 2012). Cognitively, anxiety biases attention toward threats, fosters hostile attributions, and increases the attractiveness of aggressive responses in conflict situations (Huesmann, 1988; Marsee et al., 2008). Empirical studies support this chain, showing that stress predicts anxiety, which in turn predicts externalizing or aggressive behavior (Yeo and Lee, 2017).

*Hypothesis 2:* Anxiety mediates the relationship between stress and aggressive tendencies.

### 1.3 The moderating role of “upbeat and conventional” music preference

Individual differences in music preference play a key role in stress coping. Upbeat and conventional music, characterized by positive emotional content and structured patterns, has been shown to facilitate emotion regulation (Rentfrow and Gosling, 2003). Preference for this type of music may buffer the impact of stress through two pathways: directly improving emotional states and reducing anxiety (Labbé et al., 2007), and activating prosocial cognitive scripts, thereby decreasing the accessibility of aggressive responses (Greitemeyer, 2011).

Empirical evidence supports this moderating role. Individuals favoring positive music exhibit better emotion regulation and lower aggression under stress, whereas preference for aggressive or antisocial music amplifies the stress–aggression link (Fuentes-Sánchez et al., 2022; Anderson et al., 2003). In light of the present findings and the broader literature on music-based emotion regulation, music preference may be more closely related to how stress-related and anxiety-related states are translated into aggressive tendencies than to the initial generation of anxiety itself. Accordingly, in the present study, preference for upbeat and conventional music is conceptualized as a moderator of aggression-related pathways rather than as a first-stage moderator of the stress-anxiety association. Specifically, this preference is expected to shape both the direct association between stress and aggression and the extent to which anxiety is associated with aggressive tendencies.

Hypothesis 3: Preference for “Upbeat and Conventional” music moderates the direct effect of stress on aggressive tendencies and the association between anxiety and aggressive tendencies.

### 1.4 The moderating role of perceived teacher legitimacy

Perceived teacher legitimacy represents a key social resource in school contexts and may play an important role in determining whether anxiety is translated into aggressive behavior. According to legal socialization theory, when students perceive teacher authority as legitimate, through experiences of procedural fairness and respectful treatment, they are more likely to comply voluntarily and internalize behavioral norms (Tyler, 2003; Trinkner and Tyler, 2016). Such perceptions of legitimacy are further reinforced by procedurally just practices, including transparent decision-making and respectful interpersonal treatment (Jonathan-Zamir et al., 2015).

Under anxiety, teacher legitimacy may operate through two mechanisms. First, it promotes the activation of non-aggressive, problem-solving behavioral scripts, increasing the likelihood of help-seeking, negotiation, or deferral rather than retaliation (Trinkner and Cohn, 2014). Second, it enhances students' emotion regulation and perceived social support, reducing the chance that anxiety escalates into aggression (Hazel et al., 2014). Empirical evidence indicates that high legitimacy in student–teacher relationships facilitates conciliatory responses under stress, whereas low legitimacy increases the risk that anxiety triggers aggressive cognition and behavior (Gregory and Ripski, 2008; Nivette et al., 2015). In the present study, perceived teacher legitimacy is conceptualized as a second-stage moderator. Specifically, it is expected to weaken the extent to which anxiety is translated into aggressive tendencies. Accordingly, teacher legitimacy is theorized to operate at a later stage than music preference, buffering the behavioral expression of anxiety rather than the initial emotional response to stress.

*Hypothesis 4:* Perceived teacher legitimacy negatively moderates the relationship between anxiety and aggressive tendencies, such that higher legitimacy attenuates, and lower legitimacy amplifies, the positive effect of anxiety on aggression.

### 1.5 The present study

Adopting a multidisciplinary perspective, this study integrates the General Aggression Model, music psychology, and legal socialization theory to test a moderated mediation model. Specifically, the proposed model examines whether stress predicts aggressive tendencies indirectly through anxiety, while also considering how individual and contextual factors shape aggression-related pathways. Preference for “Upbeat and Conventional” music is conceptualized as an individual emotional regulation resource that may condition the associations of stress and anxiety with aggressive tendencies, whereas perceived teacher legitimacy is conceptualized as a social-contextual factor that may weaken the effect of anxiety on aggressive tendencies. In this way, the present study seeks to clarify the distinct roles of intra-individual and contextual protective factors in the stress-anxiety-aggression process.

The hypotheses were as follows:

H1: Stress positively predicts aggressive tendencies.
H2: Anxiety mediates the relationship between stress and aggressive tendencies.
H3: Preference for “Upbeat and Conventional” music moderates the associations of stress and anxiety with aggressive tendencies.
H4: Perceived teacher legitimacy attenuates the effect of anxiety on aggressive tendencies.

Overall, this framework provides a more precise account of how individual and contextual protective factors may shape the progression from stress to anxiety and from anxiety to aggression. Accordingly, the model offers a clearer foundation for prevention programs that combine music-based emotion regulation with efforts to enhance perceived authority legitimacy in school settings.

## 2 Methods

### 2.1 Participants and procedure

Data were collected online in 2024 using the Wenjuanxing survey platform during scheduled class sessions. To ensure standardized administration and data quality, the principal investigator read a scripted set of instructions aloud in the classroom, outlining the study purpose, confidentiality procedures, and response requirements. After excluding incomplete responses, the final sample comprised 488 valid questionnaires. All participants provided informed consent prior to participation and received a small, randomly assigned monetary incentive upon completing the questionnaire, which required approximately 10 to 16 min to complete. The study protocol was approved by the Ethics Committee of Wenzhou University.

The sample characteristics were as follows. One hundred forty seven participants were male (30.1%) and 341 were female (69.9%). By year in school, 69 were first year students (14.1%), 306 were second year students (62.7%), 88 were third year students (18.0%), and 25 were fourth year students (5.1%). By major, 138 participants were enrolled in music programs (28.3%), 142 in education or psychology programs (29.1%), 164 in humanities and social sciences (33.6%), and 44 in science and engineering programs (9.0%).

### 2.2 Measures

#### 2.2.1 Music preference: the “upbeat and conventional” dimension

Music preference was measured using the “Upbeat and Conventional” dimension of the Music Preference Questionnaire (Rentfrow and Gosling, 2003). This dimension assesses preference for music that is structurally orderly, melodically clear, positively valenced, and aligned with mainstream cultural values. It comprises four genres: country, pop, religious, and soundtracks. Items were rated on a seven-point scale ranging from 1 (strongly dislike) to 7 (strongly like), with higher scores indicating stronger preference. The dimension has demonstrated acceptable reliability and validity in the original study (Rentfrow and Gosling, 2003). In the present study, internal consistency was α = 0.68. A confirmatory factor analysis further supported the acceptability of the single-factor structure, with good model fit (CFI = 0.955, SRMR = 0.034). Factor loadings ranged from 0.47 to 0.58, and composite reliability was ω = 0.66. Taken together, these findings support the use of this dimension in the present study, while also suggesting that future research may benefit from more refined measurement of music preference.

#### 2.2.2 Ultra-brief anxiety subscale of the PHQ-4

Anxiety symptoms were assessed with the two-item anxiety subscale of the Patient Health Questionnaire-4 (PHQ-4), validated for general populations by Löwe et al. (2010). The two items include, for example, “feeling nervous, anxious, or on edge.” Items were scored on a four-point scale from 0 (never) to 3 (nearly every day), with higher total scores indicating more frequent anxiety symptoms. In the current sample, the anxiety subscale demonstrated good internal consistency, α = 0.850.

#### 2.2.3 Stress subscale of the DASS-21

Perceived stress was measured using the seven-item stress subscale of the short-form Depression Anxiety Stress Scale (DASS-21), originally developed by Antony et al. (1998) and adapted into Chinese by Gong et al. (2010). Sample items include “I found it hard to relax.” Respondents rated how much each statement applied to them over the past week on a four-point scale from 0 (did not apply to me at all) to 3 (applied to me very much or most of the time). Higher scores indicate greater perceived stress. In this study the stress subscale demonstrated good internal consistency, α = 0.866.

#### 2.2.4 Brief Aggression Questionnaire (BAQ)

Trait aggression was assessed with the 12-item Brief Aggression Questionnaire (BAQ; Webster et al., 2014). Items (for example, “I might physically attack someone who angers me”) are rated on a five-point scale from 1 (extremely uncharacteristic of me) to 5 (extremely characteristic of me). A mean score across all items was computed for each participant, with higher scores indicating greater trait aggression. In the current sample, the BAQ demonstrated acceptable internal consistency, Cronbach's α = 0.770.

#### 2.2.5 Perceived teacher legitimacy

Perceived teacher legitimacy was measured using a 10-item scale adapted from the parental legitimacy measure developed by Trinkner et al. (2012) for use in the school context. Items assess students' endorsement of and voluntary compliance with teacher authority (for example, “Even if I do not like the way my teacher treats me, I should comply with the teacher's requests”). Responses were provided on a five-point scale from 1 (strongly disagree) to 5 (strongly agree). Higher total scores indicate greater perceived legitimacy of teacher authority. In the present study, the scale demonstrated acceptable internal consistency, Cronbach's α = 0.700.

### 2.3 Data analysis and common method bias

Variable normality was examined using the Shapiro-Wilk test, which showed that all variables significantly deviated from normality (*p* < 0.05). Accordingly, Spearman's rank-order correlations were used for bivariate analyses. However, the main hypothesis-testing analyses were conducted using parametric regression-based methods.

Group differences in demographic variables were tested using the Mann-Whitney U test for gender and the Kruskal-Wallis H test for year and major.

To reduce common method bias, both procedural and statistical remedies were adopted. Procedurally, anonymous administration, counterbalancing of item order, reverse-scored items, and distraction tasks between measurement stages were used to minimize potential common method variance. Statistically, Harman's single-factor test showed that the first unrotated factor accounted for 16.40% of the total variance, indicating that common method bias was unlikely to be a serious concern.

The moderated mediation model was tested using Hayes (2017) PROCESS macro (Model 65). Indirect and conditional effects were estimated with 5,000 bootstrap resamples. This OLS-based approach was used to test the hypothesized stage-specific moderated mediation model, with music preference moderating the first stage and perceived teacher legitimacy moderating the second stage.

## 3 Results

### 3.1 Demographic differences

Nonparametric tests were used to examine demographic differences. Gender differences were assessed with the Mann-Whitney U test, and year and major differences were tested with the Kruskal–Wallis H test. Results indicated a significant gender difference on the Upbeat and Conventional music preference dimension (*W* = 21,157.0, *p* = 0.006), with males scoring higher (*M* = 5.27) than females (*M* = 5.08). Perceived teacher legitimacy differed significantly by year (*H* = 19.78, *p* < 0.001) and by major (*H* = 20.79, *p* < 0.001). No other variables showed significant differences across demographic groups. Consequently, gender, year, and major were included as control variables in subsequent analyses.

### 3.2 Descriptive statistics and correlations

Descriptive statistics and Spearman correlations for the main study variables are presented in Table 1. Stress was positively associated with anxiety (*r* =0.614, *p* < 0.001) and aggressive behavior (*r* = 0.414, *p* < 0.001). Anxiety was also positively related to aggressive behavior (*r* = 0.335, *p* < 0.001). Perceived teacher legitimacy showed significant negative correlations with stress (*r*=-0.099, *p* < 0.05) and aggressive behavior (*r* = −0.140, *p* < 0.01). The Upbeat and Conventional music preference dimension was not significantly correlated with stress, anxiety, or aggression (*ps* > 0.05), suggesting that its role may operate through more complex moderating pathways.

**Table 1:** Descriptive statistics and correlation matrix of study variables (*N* = 488).

Variable	M	SD	1	2	3	4	5
1. Stress	1.74	0.54	-				
2. Anxiety	0.89	0.65	0.614^***^	-			
3. AB	2.72	0.56	0.414^***^	0.335^***^	-		
4. Music	5.14	0.95	−0.039	−0.030	0.034	-	
5. PTL	3.45	0.46	−0.099^*^	−0.083	−0.140^**^	0.114^*^	-

AB, Aggressive behavior; Music = Upbeat & Conventional music preference; PTL = Perceived teacher legitimacy.

^*^p < 0.05;

^**^p < 0.01;

^***^p < 0.001.

### 3.3 Moderated mediation analysis

A moderated mediation analysis was conducted using Hayes (2017) PROCESS Macro (Model 65). The results are presented in Table 2.

**Table 2:** Regression results of the moderated mediation model (*N* = 488).

Variable	Anxiety model			Aggression model		
	β	***SE***	***t***	β	***SE***	***t***
Constant	−0.170	0.145	−1.173	0.100	0.168	0.595
Stress	0.645	0.035	18.48^***^	0.342	0.052	6.57^***^
Anxiety	-	-	-	0.146	0.052	2.80^**^
Music	−0.008	0.035	−0.237	0.013	0.040	0.325
Stress × Music	−0.016	0.029	−0.537	0.177	0.056	3.19^**^
Anxiety × Music	-	-	-	−0.141	0.053	−2.65^**^
PT L	-	-	-	−0.124	0.041	−3.04^**^
Anxiety × PTL	-	-	-	−0.073	0.039	−1.88
Gender	−0.021	0.077	−0.267	0.179	0.088	2.04^*^
Grade	0.064	0.049	1.305	−0.086	0.056	−1.53
Major	0.017	0.037	0.472	0.012	0.042	0.293
*R* ^2^	0.423			0.259		
*F*	58.68^***^			16.68^***^		

Music= Upbeat & Conventional music preference; PTL, Perceived teacher legitimacy.

^*^p < 0.05,

^**^p < 0.01,

^***^p < 0.001.

In the anxiety model, stress significantly and positively predicted anxiety (β = 0.645, *p* < 0.001), accounting for 42.3% of its variance.

In the aggression model, the direct effect of stress on aggressive behavior was significantly moderated by preference for Upbeat and Conventional music (Stress × Music Preference: β = 0.177, *p* < 0.01). In addition, the effect of anxiety on aggression was significantly moderated by music preference (Anxiety × Music Preference: β = −0.141, *p* < 0.01), whereas the interaction between anxiety and perceived teacher legitimacy was not statistically significant (Anxiety × Teacher Legitimacy: β = −0.073, *p* = 0.061). Perceived teacher legitimacy also demonstrated a significant direct negative association with aggression (β = −0.124, *p* < 0.01), whereas gender was positively associated with aggression (β = 0.179, *p* < 0.05). Collectively, the model accounted for 25.9% of the variance in aggressive behavior.

The moderated mediation analysis indicated that the indirect effect of stress on aggressive behavior via anxiety was significantly conditioned by both *Upbeat & Conventional* music preference and teacher legitimacy. Specifically, under low music preference, the indirect effect decreased from 0.238 [95% Boot CI (0.106, 0.361)] at low teacher legitimacy to 0.141 [95% Boot CI (−0.005, 0.275)] at high teacher legitimacy. Under average music preference, the indirect effect remained significant only when teacher legitimacy was low (effect = 0.142) or at the mean level (effect = 0.094). In contrast, under high music preference, none of the indirect effects were significant (all 95% Boot CIs included zero). These conditional indirect effects are presented in Table 3.

**Table 3:** Moderated mediation analysis.

Music preference level	PTL level	Indirect effect	BootSE	BootLLCI	BootULCI
Low (-1SD)	Low (-1SD)	0.238	0.065	0.106	0.361
Low (-1SD)	Mean	0.190	0.061	0.065	0.303
Low (-1SD)	High (+1SD)	0.141	0.071	−0.005	0.275
Mean	Low (-1SD)	0.142	0.044	0.052	0.227
Mean	Mean	0.094	0.037	0.019	0.163
Mean	High (+1SD)	0.047	0.049	−0.053	0.136
High (+1SD)	Low (-1SD)	0.049	0.066	−0.085	0.178
High (+1SD)	Mean	0.003	0.060	−0.120	0.111
High (+1SD)	High (+1SD)	−0.043	0.065	−0.177	0.073

Music Preference = Upbeat & Conventional music preference. PTL = Perceived teacher legitimacy. BootSE = bootstrap standard error; BootLLCI = bootstrap lower limit confidence interval; BootULCI = bootstrap upper limit confidence interval. Confidence intervals not containing zero indicate statistically significant effects at p < 0.05.

Simple slope analyses indicated that music preference for “Upbeat and Conventional” styles significantly moderated the direct effect of stress on aggressive behavior. As individuals' preference for this type of music increased, the predictive effect of stress on aggression strengthened, with simple slopes rising from *b* = 0.165, *p* < 0.05 at low preference to *b* = 0.520, *p* < 0.001 at high preference.

For the latter part of the indirect path, music preference significantly moderated the effect of anxiety on aggression, whereas the interaction between anxiety and perceived teacher legitimacy did not reach statistical significance. Under low levels of “Upbeat and Conventional” music preference, anxiety positively predicted aggression, whereas under high levels of music preference, anxiety did not significantly predict aggression.

Overall, these findings support a moderated mediation model in which stress influences aggression both directly and indirectly through anxiety. The indirect pathway through anxiety was conditioned primarily by music preference, and the direct effect of stress on aggression was also contingent on music preference.

## 4 Discussion

### 4.1 the association between stress and aggressive tendencies

The present study found that stress was significantly and positively associated with aggressive tendencies, such that higher levels of stress were associated with stronger expressions of aggression. This finding aligns with both theoretical frameworks and empirical evidence. According to the stress-adaptation model (Cohen et al., 1997; Hobfoll, 2001), when environmental demands exceed an individual's coping resources, sustained or intense stress can elicit adverse emotional and physiological responses, undermining self-regulation and increasing the risk of externalizing behaviors. At the neurocognitive level, chronic stress impairs prefrontal cortex functioning, particularly in regions involved in impulse control and strategic decision-making (Arnsten, 2015; Cohen et al., 2012), rendering individuals more prone to impulsive or hostile reactions when confronted with provocation or conflict. Social psychological research further indicates that stress is linked to a greater likelihood of aggressive behavior and may also exacerbate negative interpretations of others' intentions by activating threat-related attribution patterns and hostile cognitive processing (Huesmann and Guerra, 1997; Huesmann, 2018).

Evidence from educational and adolescent development research similarly supports a robust link between stress and aggression. Academic, social status, and family-related stressors have been shown to predict aggressive or antisocial behaviors among adolescents and college students (Jun and Choi, 2015; Gao and Hu, 2013). These findings suggest that during critical developmental periods, high and persistent stress may not only impair emotional regulation but also lower behavioral inhibition thresholds and reinforce hostile response patterns, thereby facilitating aggressive expression. Overall, the present results support the view that stress is an important correlate of aggressive tendencies, highlighting the potential value of stress management and coping resources in aggression prevention.

### 4.2 The mediating role of anxiety

The present study showed that anxiety statistically mediated the association between stress and aggression, providing empirical support for an indirect association between stress and aggressive tendencies via anxiety (Marsee et al., 2008; Yeo and Lee, 2017), thereby supporting Hypothesis 2. From an emotion–cognition perspective, anxiety, a high-arousal negative emotional state, consumes cognitive resources, heightens attention to threat cues, and promotes emotion-driven decision-making (Hardin et al., 2007; Robinson et al., 2013). These processes increase the likelihood that individuals will adopt hostile or avoidant response strategies when confronted with provocation or conflict, ultimately manifesting in aggressive behavior. Empirical evidence further indicates that social or emotional stress elevates anxiety levels, which in turn is associated with externalizing problems (Paulus et al., 2021; Yang et al., 2023).

### 4.3 The differential roles of music preference and teacher legitimacy

The present findings suggest that music preference and perceived teacher legitimacy were associated with different patterns in the stress-anxiety-aggression process. Preference for “Upbeat and Conventional” music significantly moderated both the direct effect of stress on aggression and the association between anxiety and aggression. Specifically, higher preference for this music style was associated with a stronger direct effect of stress on aggression, but a weaker effect of anxiety on aggression. In contrast, perceived teacher legitimacy was negatively associated with aggression at the main-effect level, but its interaction with anxiety did not reach statistical significance. Thus, the results support a differentiated pattern in which music preference functioned as a significant moderator, whereas teacher legitimacy appeared to operate primarily as a broader contextual protective factor.

This pattern is consistent with a person-situation perspective on aggression. The General Aggression Model suggests that aggressive behavior reflects the joint influence of person-level dispositions and situational inputs on internal states and behavioral outcomes. Within this framework, music preference may be understood as an intra-individual regulatory tendency, whereas perceived teacher legitimacy reflects the normative and relational quality of the school environment. Prior research indicates that music is frequently used for emotion regulation, but its effects are not uniformly adaptive and may vary depending on listening motives, regulatory style, and context (Carlson et al., 2015; Saarikallio, 2011; Saarikallio et al., 2015). Accordingly, the present findings suggest that preference for “Upbeat and Conventional” music may be involved in stress-related processes in more than one way. On the one hand, it was associated with a weaker anxiety-aggression association, suggesting that it may help reduce the behavioral carryover of anxiety. On the other hand, its strengthening of the direct stress-aggression path indicates that its role is not uniformly protective. Because the current data do not assess actual music use, listening motives, or situational exposure, the mechanism underlying this counterintuitive direct effect should be interpreted cautiously and warrants further investigation. Overall, this pattern should be regarded as a preliminary and exploratory finding.

The findings for perceived teacher legitimacy add an important social-contextual component to the model. Although teacher legitimacy did not significantly moderate the anxiety–aggression link, its negative association with aggression is consistent with legal socialization and procedural justice perspectives, which emphasize that fair, respectful, and trustworthy authority promotes norm internalization and reduces oppositional or aggressive responding (Tyler, 2003; Trinkner and Tyler, 2016). Research on school fairness and legitimacy similarly shows that students' perceptions of just and respectful teacher treatment are associated with stronger legitimacy beliefs and lower aggression or misconduct (Gouveia-Pereira et al., 2017; Gini et al., 2024). In this sense, teacher legitimacy may not have altered the strength of the anxiety-aggression path in a statistically robust way in the present study, but it was associated with a lower overall likelihood of aggressive responding within the school context.

Taken together, these findings provide a more integrative account of how intra-individual and social-contextual factors may jointly relate to aggression. Music preference appears to matter most in shaping how stress-related emotional processes unfold, whereas teacher legitimacy appears to matter more in defining a lower-risk interpersonal climate overall. This distinction helps refine the person–situation framework proposed in the Introduction by showing that individual and contextual protective factors may both be relevant, while operating through different empirical pathways.

### 4.4 Overall evaluation, limitations, and future directions

This study used a moderated mediation framework to examine the associations among stress, anxiety, and aggressive tendencies. The findings suggest that stress was associated with aggression both indirectly through anxiety and directly through a pathway moderated by music preference. Music preference also moderated the anxiety-aggression association, whereas perceived teacher legitimacy was negatively associated with aggression at the main-effect level but did not significantly moderate the anxiety-aggression link. Taken together, these findings underscore the relevance of both intra-individual regulatory tendencies and social-contextual resources in understanding aggression, and they extend the General Aggression Model by incorporating music preference and perceived authority legitimacy in school-aged and young adult populations (Anderson and Bushman, 2002; Bartett et al., 2021).

Several limitations should be acknowledged. First, the cross-sectional design does not permit causal inference or firm conclusions about temporal ordering. Second, the internal consistency of the “Upbeat and Conventional” music preference dimension was relatively modest, which may have reduced statistical power. Third, self-reported music preference did not capture listening motives, situational exposure, or content-specific features such as lyrical aggression or prosociality. Fourth, perceived teacher legitimacy was measured subjectively, which may limit external validity.

Future research should use longitudinal, experimental, and multi-method designs to clarify the temporal dynamics of the stress–anxiety–aggression process. More refined assessments of music-related processes, including listening motives, emotion-regulation strategies, and contextual exposure, are also needed. Improving the reliability of music preference measures, for example by adding items or using more developed instruments such as the short version of the STOMP, may strengthen future analyses. Finally, school-based intervention studies should examine whether combining procedural justice approaches with emotion-regulation strategies can help reduce aggressive tendencies.

## 5 Conclusion

This study elucidates the interactive mechanisms through which stress, anxiety, music preference, and perceived teacher authority contribute to the development of aggressive behavior. Stress exerts both a direct effect on aggression and an indirect effect through anxiety, with the latter half of the mediating pathway buffered by “Upbeat and Conventional” music preference and teacher authority. In the direct pathway, music preference amplifies the impact of stress, whereas in the mediated pathway, music preference and teacher authority jointly attenuate the influence of anxiety on aggression.

The findings extend a multidisciplinary understanding of aggressive behavior by integrating individual emotional regulation through music and contextual educational factors. They also offer practical implications: enhancing teacher legitimacy and aligning interventions with students' music preferences and emotion management strategies may effectively mitigate aggression under stress.

## Data Availability

The raw data supporting the conclusions of this article will be made available by the authors, without undue reservation.
